# Acamprosate reduces ethanol intake in the rat by a combined action of different drug components

**DOI:** 10.1038/s41598-023-45167-3

**Published:** 2023-10-19

**Authors:** Karin Ademar, Anna Loftén, Mathilda Nilsson, Ana Domi, Louise Adermark, Bo Söderpalm, Mia Ericson

**Affiliations:** 1 Addiction Biology Unit, Department of Psychiatry and Neurochemistry, Institute of Neuroscience and Physiology, The Sahlgrenska Academy, University of Gothenburg, Box 410, 405 30 Gothenburg, Sweden; 2 Department of Pharmacology, Institute of Neuroscience and Physiology, The Sahlgrenska Academy, University of Gothenburg, Gothenburg, Sweden; 3 Beroendekliniken, Sahlgrenska University Hospital, Gothenburg, Sweden

**Keywords:** Neural circuits, Neuronal physiology, Reward

## Abstract

Alcohol misuse accounts for a sizeable proportion of the global burden of disease, and Campral^®^ (acamprosate; calcium-bis-(*N*-acetylhomotaurinate)) is widely used as relapse prevention therapy. The mechanism underlying its effect has in some studies been attributed to the calcium moiety and not to the *N*-acetylhomotaurine part of the compound. We recently suggested that the dopamine elevating effect of acamprosate is mediated both by *N*-acetylhomotaurine and calcium in a glycine receptor dependent manner. Here we aimed to explore, by means of in vivo microdialysis, if our previous study using local administration was functionally relevant and if systemic administration of the sodium salt of *N*-acetylhomotaurine (sodium acamprosate; 200 mg/kg, i.p.) enhanced the effects of calcium chloride (CaCl_2_; 73.5 mg/kg, i.p.) on nucleus accumbens (nAc) dopamine and/or taurine levels in male Wistar rats. In addition, we investigated the impact of regular acamprosate and the combination of CaCl_2_ and *N*-acetylhomotaurine on the alcohol deprivation effect (ADE). Finally, we assessed if *N*-acetylhomotaurine potentiates the ethanol-intake reducing effect of CaCl_2_ in a two-bottle choice voluntary ethanol consumption model followed by an ADE paradigm. Systemic administration of regular acamprosate, sodium acamprosate and CaCl_2_ all trended to increase nAc dopamine whereas the combination of CaCl_2_ and sodium acamprosate produced a significant increase. Sodium acamprosate elevated extracellular taurine levels without additional effects of CaCl_2_. Ethanol intake was significantly reduced by systemic administration of CaCl_2_ without additional effects of the combination of CaCl_2_ and sodium acamprosate. Both acamprosate and CaCl_2_ combined with sodium acamprosate blocked the ADE following acute treatment. The data presented suggest that CaCl_2_ and *N*-acetylhomotaurine act in concert on a neurochemical level, but calcium appears to have the predominant effect on ethanol intake.

## Introduction

Acamprosate (calcium-bis(*N*-acetylhomotaurinate)), comprises of two *N*-acetylhomotaurine molecules linked to a calcium ion and is one of few pharmacotherapies available for treatment of alcohol use disorder (AUD)^1^. AUD is a neuropsychiatric disorder that involves several neuronal pathways, and a well-functioning treatment is both fundamental and challenging. The difficulty of finding an ideal treatment strategy probably originates from i.e. the heterogeneity of patients diagnosed with AUD and the limited number of currently accessible drugs^2^. Furthermore, the number needed treat (NNT) is relatively high and the effect sizes of AUD treatments are generally low^3^. The NNT of acamprosate has been estimated to 9–12 for preventing return to any drinking (Cochrane review of 24 trials, n = 6915^4^; meta-analysis of 27 studies, n = 7519^3^).

Acamprosate, in distinction to other approved pharmacotherapies such as naltrexone, nalmefene and disulfiram, lacks an established molecular mode of action. Previous studies have suggested that acamprosate interacts with both inhibitory and excitatory neurotransmission to restore the ethanol-induced imbalance between glutamatergic and GABAergic transmission^5–7^. In addition, acamprosate has been shown to increase dopamine in the mesolimbic dopamine system^8–10^, an effect postulated to partly mimic ethanol and to be mediated by a mesolimbic neuronal circuitry involving glycine receptor (GlyR) activation in the nucleus accumbens (nAc)^9,11^. Contravening early discoveries, the effects of acamprosate have lately been suggested to be mediated primarily by the calcium ion linking the two *N-*acetylhomotaurine molecules^12^. In fact, calcium salts were found to have acamprosate-like effects in animal models of ethanol craving and relapse^12^. Indeed, acamprosate and calcium alone were demonstrated to reverse ethanol-induced cognitive deficits in mice^13^, whereas the sodium salt of *N*-acetylhomotaurine had no effect^13^. These studies concluded that calcium is the active component of acamprosate.

GlyRs in the nAc were previously shown to be of importance both for the dopamine elevating properties of acamprosate and for its ethanol-intake reducing effect, as local application of strychnine into the nAc reversed both effects^9,14^. Recently, we found calcium chloride to elevate dopamine in a similar GlyR sensitive manner and that calcium chloride combined with sodium acamprosate produced an additive increase in dopamine when applied locally in the nAc^15^. Acamprosate has also been shown to increase nAc taurine levels^15,16^, providing the possibility for an indirect activation of GlyRs.

While the therapeutic role of acamprosate is clear, its pharmacological mechanism of action needs to be established in more detail to further the understanding of targets that interfere with AUD. Since we recently found *N*-acetylhomotaurine and calcium to act in combination with regards to nAc dopamine and taurine following local administration in nAc, we wanted to continue investigating this combination for functional relevance following systemic application. In the present study, we used in vivo microdialysis to explore nAc dopamine and taurine responses following systemic administration of calcium chloride, different salts of *N*-acetylhomotaurine and the combination of calcium chloride and sodium acamprosate. We also investigated if regular acamprosate and calcium chloride in combination with sodium acamprosate are equally efficient in preventing the alcohol deprivation effect and if sodium acamprosate influences the ethanol intake reducing effects of calcium chloride.

## Material and methods

### Animals

Male Wistar rats (Envigo, the Netherlands) were used in the study. For in vivo microdialysis, 54 animals at age 9–10 weeks weighing 290–370 g were group-housed at regular light–dark conditions (lights on at 7:00 A.M. and lights off at 7:00 P.M.), constant room temperature (20–22 °C) and humidity (55–65%). For voluntary ethanol consumption, 80 rats at age 6–7 weeks weighing 170–220 g at the initiation of the study were single-housed (cage size: 40 × 24 × 18 cm) at reversed light–dark conditions (lights on at 10:00 PM and lights off at 10:00 A.M.), constant room temperature (20–22 °C) and humidity (50–55%). Throughout the entire experiments, animals had continuous access to standard rodent chow and water ad libitum. At arrival to the animal facilities, animals were allowed one week of acclimatization to the new environment, while being group-housed (cage size: 55 × 35 × 20 cm), before any experiments started. All experiments in the study are approved by the Ethics Committee for Animal Experiments, Gothenburg, Sweden, and conducted in accordance with the relevant guidelines. The study is reported in accordance with ARRIVE guidelines.

### Drugs and solutions

Calcium-bis-(*N*-acetylhomotaurinate) (acamprosate/CaAcamp) (kindly provided by Merck, Lyon, France), sodium-*N*-acetylhomotaurinate (sodium acamprosate/NaAcamp) (TCI, Zwijndrecht, Belgium) and CaCl_2_ $*$ 2H_2_O (Fisher Scientific, Gothenburg, Sweden) were all dissolved in 0.9% NaCl. Ethanol (95% Kiilto Clean AB, Täby, Sweden) was diluted in tap water. All drugs were administered systemically (i.p.) in a volume of 2 ml/kg, while the ethanol solution was added to the home cage fluid bottles.

### Experimental design

Three separate experiments were performed on drug naïve animals. In the in vivo microdialysis study, extracellular accumbal dopamine and taurine levels were monitored in awake and freely moving animals for 180 min following acute treatment with vehicle (0.9% NaCl, i.p.), CaAcamp (200 mg/kg, i.p.), NaAcamp (200 mg/kg, i.p.), CaCl_2_ (73.5 mg/kg, i.p.) or the combination of CaCl_2_ and NaAcamp.

In the first voluntary ethanol consumption study we monitored the alcohol deprivation effect (ADE) following administration of vehicle (0.9% NaCl, i.p.) and aimed to repeat previous studies showing inhibition of ADE following treatment with CaAcamp (200 mg/kg, i.p.) or the combination of CaCl_2_ (73.5 mg/kg, i.p.) and NaAcamp (200 mg/kg, i.p.). In the second voluntary ethanol consumption study, a limited access paradigm with 6-h daily access to ethanol was used to evaluate if the addition of NaAcamp enhanced the ethanol-intake reducing effects of CaCl_2_. Separate groups of animals received daily injections with vehicle (0.9% NaCl, i.p.), CaCl_2_ (73.5 mg/kg, i.p.) or the combination of CaCl_2_ (73.5 mg/kg, i.p.) and NaAcamp (200 mg/kg, i.p.) 30 min before accessing the ethanol bottle. Ethanol and water intake were calculated as (g/kg/6 h during the treatment phase or 24 h during the screening phase) and preference for ethanol as (% ml ethanol solution/ml total fluid intake per 6 h or 24 h; schematic overview of experimental design in Fig. 2).

The doses used for the in vivo microdialysis experiments and the ethanol consumption studies were based on 200 mg/kg of CaAcamp that previously was shown to decrease ethanol intake in a robust manner^14,17^. For the CaCl_2_ treatment, 73.5 mg/kg was used to consider equivalent amounts of Ca^2+^-ions (0.499 mmol/kg) injected. Further, the amount of *N*-acetylhomotaurine in 200 mg/kg CaAcamp and 200 mg/kg NaAcamp was practically the same differing with 1.46% (0.999 mmol/kg and 0.984 mmol/kg respectively).

### In vivo microdialysis

Two days prior to the experiment, rats were surgically equipped with a dialysis probe. In brief, rats were anesthetized with 4% isoflurane (Baxter, Kista, Sweden) and placed on a heating pad while being mounted onto a stereotaxic instrument (David Kopf Instruments, AgnTho’s, Lidingö, Sweden). The skull was exposed, and one hole was drilled for the probe placement and two holes for anchoring screws. The custom-made dialysis probe, with an active space of 2 mm and a molecular cut-off of 20 kDa, was unilaterally lowered into the nAc core–shell borderline region (A/P: + 1.85 mm, M/L: − 1.4 mm relative to bregma, D/V: − 7.8 mm relative to dura^18^). The anchoring screws and the probe were fixed to the skull by using Harvard cement (DAB Dental AB, Gothenburg, Sweden). Animals were single-housed during the 48-h surgical recovery.

**Figure 1:**
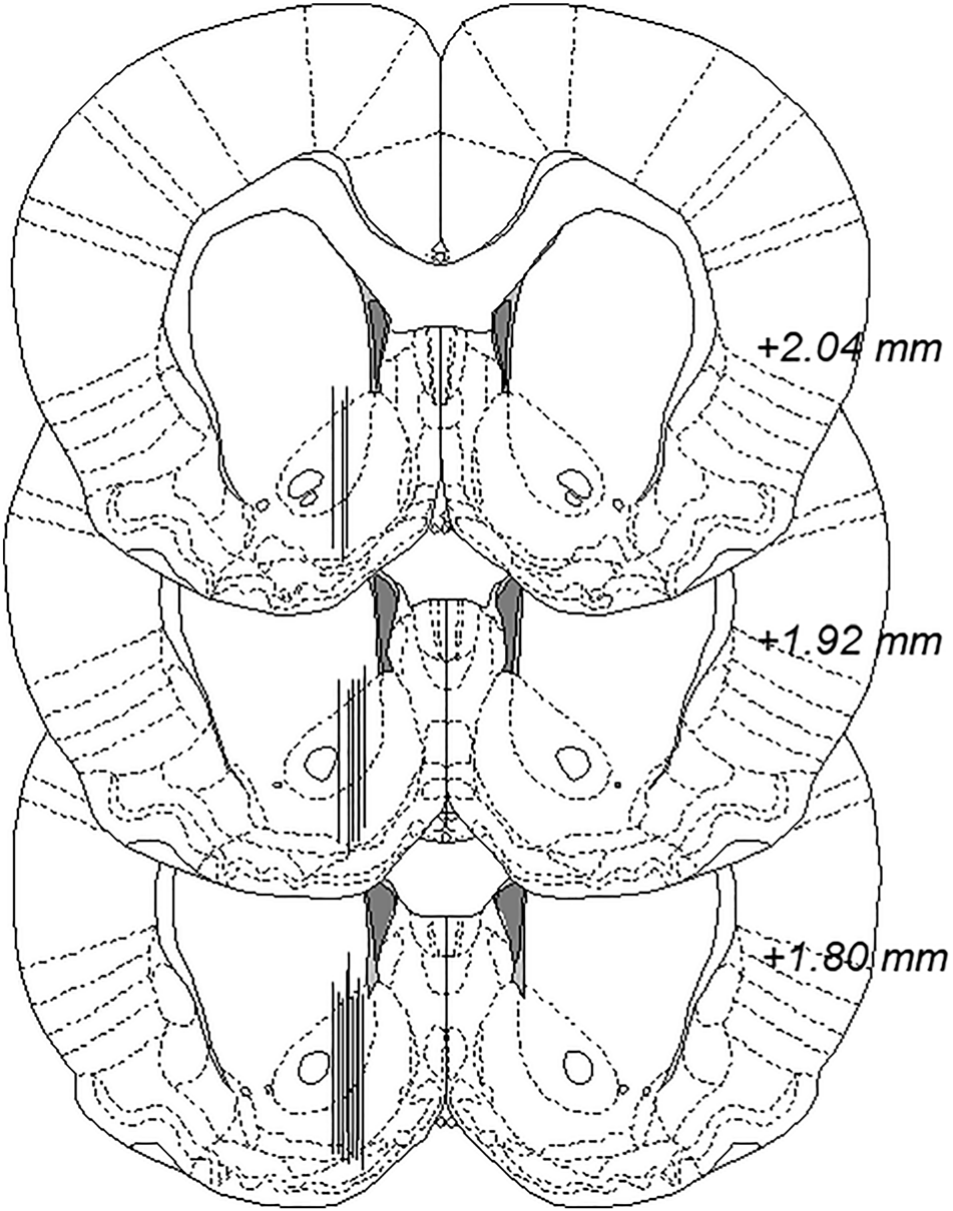
Histology. Representation of microdialysis probe placement in the nAc. The black lines illustrate traces of approximately every second animal included in the study. Adjacent numbers indicate distance from bregma.

On the experimental day, the inlet and outlet of probes were cut open and connected to a microperfusion pump (U-864 Syringe Pump, AgnTho’s, Lidingö, Sweden) via a swivel allowing the rat to move around freely in its home cage. Animals with a postoperative weight loss (more than 10% of the preoperative weight) were excluded. Throughout the dialysis experiment, the probe was perfused with Ringer’s solution (140 NaCl, 1.2 CaCl_2_, 3.0 KCl and 1.0 MgCl_2_ (all in mmol/l)) at a rate of 2 µl/min and dialysate samples (40 µl) were collected every 20 min. In order to obtain balance in the fluid exchange over the dialysis membrane, the rats were perfused with Ringer’s solution for 2 h before baseline sampling was initiated. The acute pharmacological treatment was administered when a stable baseline with regards to dopamine levels (± 10%) was obtained. Animals were sacrificed instantly after the experiment, the brains were removed and placed in fixative (Accustain, Sigma-Aldrich, Sweden) for 4–7 days until probe placement verification using a vibroslicer (Campden Instruments Ltd, Leicester, UK). Visual brain tissue damage or misplaced probes excluded animals from statistical analysis (see Fig. 1 for probe placements).

For the analysis of dopamine and taurine, each dialysate sample was split and analyzed separately. Different high-performance liquid chromatography (HPLC) systems, coupled with electrochemical or fluorescent detection, were used for the separation and detection of dopamine and taurine as previously described^19,20^. For the online identification and quantification of the dopamine peak, an external standard containing 3.25 nM of dopamine was used. Two external standards containing 500 or 1000 nM taurine were used to identify and quantify the taurine peak. To each individual taurine sample sodium azide (50% v/v) was added to maintain stability of the samples. All chromatograms were analyzed using Thermo Scientific Chromeleon Chromatography Data System (CDS) software (CHROMELEON7). A total of 7 animals were excluded from the microdialysis study.

### Voluntary ethanol consumption

**Figure 2:**
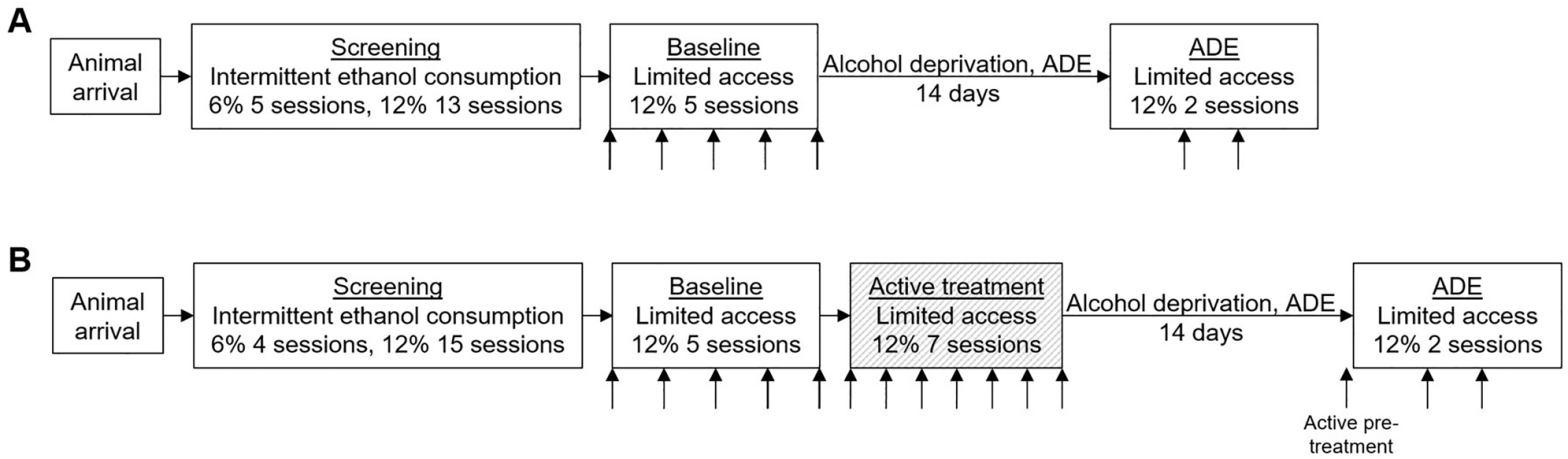
Schematic overview of experimental design. The experimental design of the voluntary ethanol consumption studies monitoring (**A**) acute treatment on ADE and (**B**) limited access paradigm with repeated drug exposure followed by ADE. Arrows indicate vehicle or drug injections. *ADE* alcohol deprivation effect.

Rats (n = 30) were placed on an intermittent voluntary ethanol consumption paradigm, with access to ethanol for three 24-h sessions per week (bottles were added on Monday, Wednesday and Friday). The rats were introduced to a bottle containing 6% (v/v) ethanol, which after two weeks, was increased to contain 12% (v/v) ethanol for an additional five weeks. After the seven-week period of intermittent ethanol consumption, animals consuming less than 1.5 g/kg/24 h during the last three drinking sessions were excluded. A total of 27 rats were placed on a limited access paradigm with access to the ethanol bottle six hours daily, starting at the beginning of the dark period. As preparation for the pharmacological treatment, an injection of saline was administered each day for 5 days (baseline) before the bottle of 12% ethanol was presented for the upcoming 6 h. Rats were then evenly assigned to three treatment groups (vehicle, 0.9% NaCl; CaAcamp, 200 mg/kg; CaCl_2_ + NaAcamp, 73.5 mg/kg + 200 mg/kg), based on the average ethanol consumption during the days of saline treatment, and exposed to 14 days of alcohol deprivation with free access to water but no ethanol. Following the deprivation period, each animal was systemically injected (i.p.) for two days with the assigned pharmacological treatment. Ethanol was reintroduced on the first day of injections (Fig. 2A).

In the second voluntary ethanol consumption study, 50 rats were screened as described above. After the seven-week period of intermittent ethanol consumption, animals consuming less than 1.5 g/kg/24 h during the last three drinking sessions were excluded. A total of 43 rats were placed on a limited access paradigm, with ethanol (12%) access for the upcoming 6 h starting at the beginning of the dark period. Animals were administered saline each day for 5 days (baseline) before accessing the ethanol bottles and were in the subsequent step evenly assigned to three treatment groups based on the average ethanol consumption during the days of saline treatment. Active treatment (CaCl_2_, 73.5 mg/kg or CaCl_2_ + NaAcamp, 73.5 mg/kg + 200 mg/kg) or vehicle (0.9% NaCl) was systemically injected 30 min before accessing the daily 6-h drinking session for 7 days. Following the pharmacological treatment, rats were exposed to 14 days of alcohol deprivation with free access to water but no ethanol. Each animal was then subjected to another three injections of pharmacological treatment, while the ethanol bottles were reintroduced on the second day of injections (Fig. 2B). Based on other studies^12^, we did not expect the sodium salt of *N*-acetylhomotaurine alone to show efficacy on ethanol intake and did not include this treatment group in the experiment. During the entire experiments, the animals were observed daily, and the body weight was measured once a week.

### Statistics

The microdialysis and ethanol consumption data were statistically evaluated using GraphPad Prism 9 Software (San Diego, CA, USA). For the microdialysis experiments, data were statistically evaluated using a two-way analysis of variance (ANOVA) with repeated measures (treatment group x time) followed by Dunnett’s multiple comparisons test. For the ethanol consumption studies, paired *t*-test and a one-way ANOVA was used for analysis of the ADE and a two-way ANOVA with repeated measures (treatment group × time) followed by Tukey’s multiple comparisons test was used for statistical evaluation of the limited access paradigm. All data are expressed as means ± standard error of the mean (SEM) and statistical significance considered a probability value (*p*) less than 0.05.

## Results

### Enhanced accumbal dopamine levels after systemic administration with the combination of calcium chloride and sodium acamprosate

**Figure 3:**
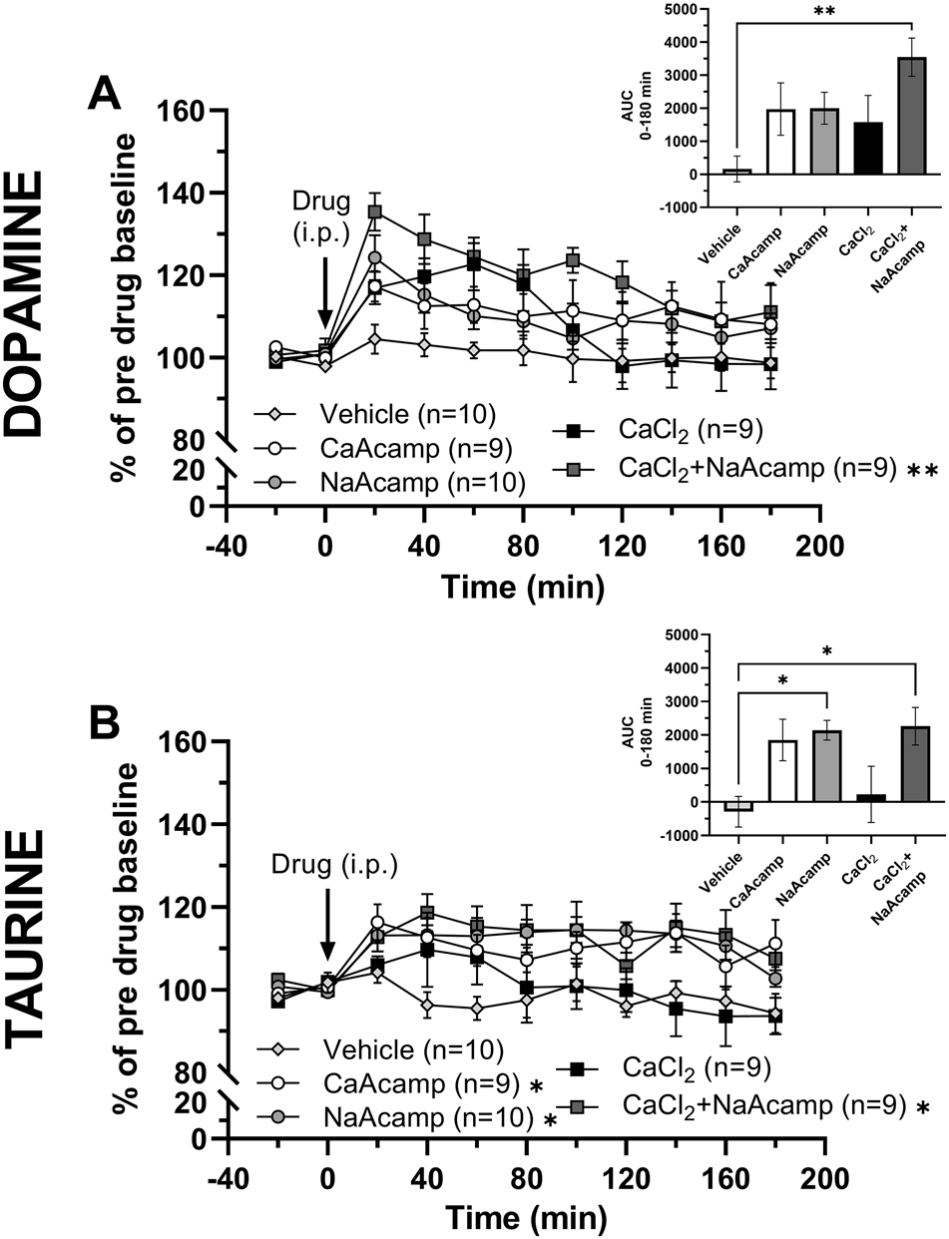
Combined administration of calcium chloride and sodium acamprosate elevates extracellular accumbal dopamine levels in an enhanced manner, and raises taurine levels. In vivo microdialysis time-course graphs of the rat nucleus accumbens (**A**) dopamine and (**B**) taurine levels following systemic administration (i.p.) of CaAcamp (200 mg/kg; n = 9), NaAcamp (200 mg/kg; n = 10), CaCl_2_ (73.5 mg/kg; n = 9) and CaCl_2_ + NaAcamp (73.5 mg/kg + 200 mg/kg; n = 9). A significant increase of dopamine levels was produced by the combination of CaCl_2_ + NaAcamp (**A**), while taurine levels were elevated by CaAcamp, NaAcamp and CaCl_2_ + NaAcamp (**B**). Inserts represents area under the curve for dopamine (**A**) and taurine (**B**). Arrows indicate time point for drug administration. * denotes a significant result compared to vehicle. All data are presented as mean ± SEM. *p < 0.05, **p < 0.01. *AUC* area under the curve, *CaAcamp* calcium acamprosate, *CaCl*_*2*_ calcium chloride, *NaAcamp* sodium acamprosate.

Dopamine levels were monitored over time in the nAc during systemic administration of either vehicle, CaAcamp (200 mg/kg, i.p.), NaAcamp (200 mg/kg, i.p.) or CaCl_2_ (73.5 mg/kg, i.p.), alone or in combination. In vivo microdialysis experiments demonstrated a significant effect by treatment on extracellular dopamine levels (two-way ANOVA with repeated measures_t=0–180 min_: treatment effect F_(4,42)_ = 3.55, *p* = 0.014; time effect F_(9,378)_ = 12.62, *p* < 0.001; treatment*time interaction F_(36,378)_ = 1.61, *p* = 0.017; Fig. 3A). While a visual trend for elevated dopamine levels was observed for all treatments, only rats treated with the combination of CaCl_2_ and NaAcamp demonstrated a significant increase in dopamine compared to vehicle-treated controls (Dunnett’s post hoc test: vehicle vs CaAcamp *p* = 0.152, vehicle vs NaAcamp *p* = 0.204, vehicle vs CaCl_2_ *p* = 0.373, vehicle vs CaCl_2_ + NaAcamp *p* = 0.002; Fig. 3A). Evaluation of dopamine levels using area under the curve (AUC) for the treatment period demonstrated a similar statistical outcome (one-way ANOVA of AUC_t=0–180 min_: F_(4, 42)_ = 3.85, *p* = 0.009; Tukey’s post hoc test: vehicle vs CaAcamp *p* = 0.248, vehicle vs NaAcamp *p* = 0.212, vehicle vs CaCl_2_ *p* = 0.489, vehicle vs CaCl_2_ + NaAcamp *p* = 0.003; Fig. 3A insert).

The extracellular levels of taurine were investigated in the nAc of the same animals. Systemic drug administration produced a significant effect of treatment on taurine levels in the nAc (two-way ANOVA with repeated measures_t=0–180 min_: treatment effect F_(4,42)_ = 4.29, *p* = 0.005; time effect F_(9,378)_ = 4.76, *p* < 0.001; treatment*time interaction F_(36,378)_ = 1.51, *p* = 0.034; Fig. 3B). Post hoc analysis demonstrated that taurine was significantly increased by all treatments containing the *N*-acetylhomotaurine molecule (Dunnett’s post hoc test: vehicle vs CaAcamp *p* = 0.034, vehicle vs NaAcamp *p* = 0.016, vehicle vs CaCl_2_ *p* = 0.935, vehicle vs CaCl_2_ + NaAcamp *p* = 0.011; Fig. 3B). Exploring AUC for extracellular taurine revealed increased taurine levels following NaAcamp treatment (one-way ANOVA of AUC_t=0–180 min_: F_(4, 42)_ = 4.39, *p* = 0.005; Tukey’s post hoc test: vehicle vs CaAcamp *p* = 0.075, vehicle vs NaAcamp *p* = 0.026, vehicle vs CaCl_2_ *p* = 0.967, vehicle vs CaCl_2_ + NaAcamp *p* = 0.022; Fig. 3B insert).

### ADE is prevented by both regular acamprosate and calcium combined with sodium acamprosate

**Figure 4:**
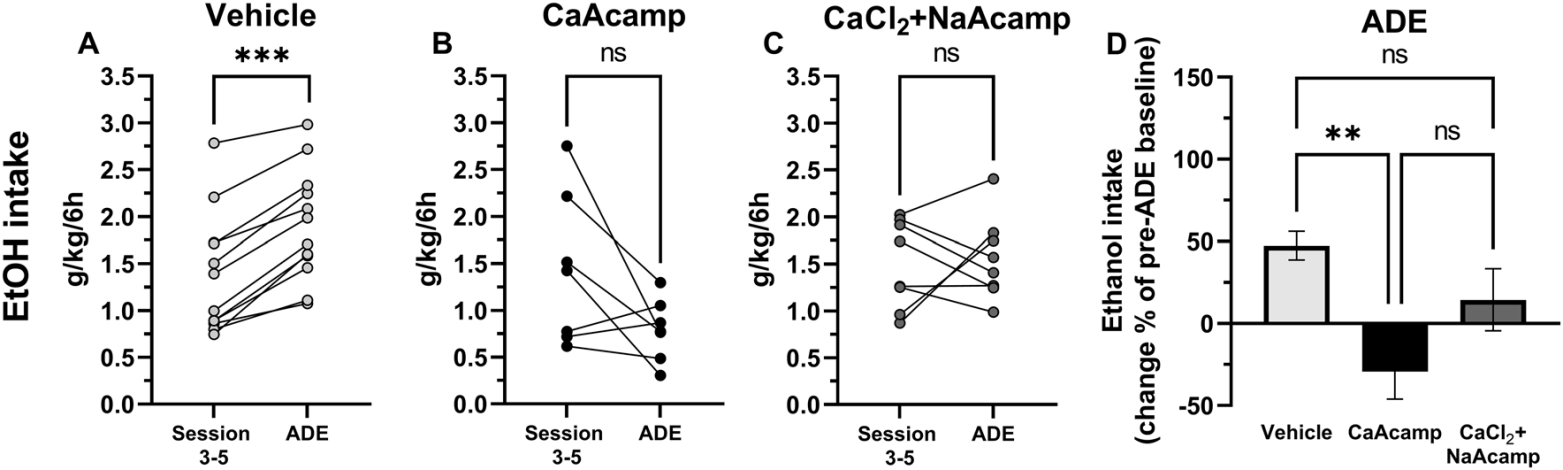
Treatment with calcium acamprosate and the combination of calcium chloride and sodium acamprosate prevent the alcohol deprivation effect. Mean ethanol intake (g/kg/6 h) of the last three sessions of the baseline period compared with the mean of two sessions following two weeks of ethanol deprivation (**A**–**D**). A significant increase in ethanol intake was seen in vehicle (0.9% NaCl; n = 12) treated animals (**A**), but not in animals treated with CaAcamp (200 mg/kg; n = 7) (**B**) or CaCl_2_ + NaAcamp (73.5 mg/kg + 200 mg/kg; n = 8) (**C**). Comparison on a group level showed a decreased ethanol consumption following CaAcamp as compared to vehicle (**D**). **p < 0.01, ***p < 0.001. *ADE* alcohol deprivation effect, *CaAcamp* calcium acamprosate, *CaCl*_*2*_ calcium chloride, *NaAcamp* sodium acamprosate.

Since we and others previously found a loss of the ethanol-intake reducing effect of regular acamprosate following repeated administration^17,21^, a phenomenon linked to the drug’s ability to increase dopamine^22^, we aimed to target the influence of acute drug treatment with CaAcamp as well as CaCl_2_ in combination with NaAcamp on the ADE in a separate study. CaCl_2_ alone has previously been demonstrated to block the ADE following acute treatment^12^. When reintroducing the 6-h limited access to ethanol (12%, v/v) for two days following the two consecutive weeks of abstinence, animals receiving vehicle treatment displayed an increased ethanol consumption compared with consumption prior to ethanol deprivation (mean of the last three treatment sessions) (paired *t*-test: t_(11)_ = 8.56, *p* < 0.001; Fig. 4A). No significant difference in ethanol consumption following ethanol deprivation was seen in animals receiving CaAcamp (paired *t*-test: t_(6)_ = 2.11, *p* = 0.080; Fig. 4B) or CaCl_2_ in combination with NaAcamp (paired *t*-test: t_(7)_ = 0.286, *p* = 0.783; Fig. 4C). When comparing the change in alcohol intake in the three groups following the alcohol deprivation period, animals receiving CaAcamp demonstrated a decreased alcohol consumption compared to vehicle-treated rats (one-way ANOVA: F_(2, 24)_ = 7.593, *p* = 0.003; Tukey’s post hoc test: vehicle vs CaAcamp *p* = 0.002; Fig. 4D).

### Voluntary ethanol consumption is stably reduced by combined calcium chloride and sodium acamprosate administration

**Figure 5:**
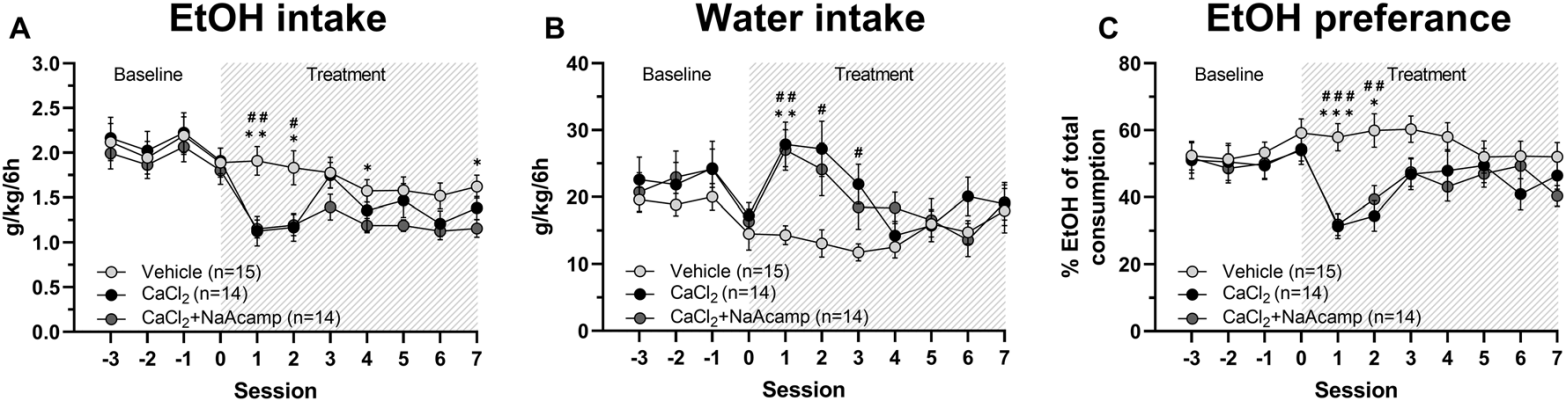
Calcium chloride combined with sodium acamprosate produce a prolonged reduction of ethanol intake. Systemic injection (i.p.) of CaCl_2_ (73.5 mg/kg; n = 14) as well as CaCl_2_ + NaAcamp (73.5 mg/kg + 200 mg/kg; n = 14) significantly decreased ethanol intake (g/kg/6 h) during the first two treatment sessions, as compared to vehicle treated controls (n = 15; **A**), while a prolonged reduction in ethanol intake was induced solely by CaCl_2_ + NaAcamp (**A**). Water intake (g/kg/6 h) (**B**) and ethanol preference (% EtOH of total consumption) (**C**) demonstrated a significant escalation and reduction, respectively, throughout the treatment sessions, as compared with vehicle controls. # denotes CaCl_2_ significant compared to vehicle and * denotes CaCl_2_ + NaAcamp significant compared to vehicle. All data are presented as mean ± SEM. *p < 0.05, **p < 0.01, ***p < 0.001. *CaCl*_*2*_ calcium chloride, *EtOH* ethanol, *NaAcamp* sodium acamprosate.

In the second voluntary ethanol consumption study, possible enhanced effects produced by the combination of CaCl_2_ and NaAcamp, as compared to CaCl_2_ alone, were examined with regards to ethanol and water consumption (see Fig. S1 for screening data). A two-way ANOVA with repeated measures revealed a significant effect of treatment (F_(2,40)_ = 4.26, *p* = 0.021), time (F_(5.96,238)_ = 9.65, *p* < 0.001) and treatment*time interaction (F_(14,280)_ = 2.16, *p* = 0.009) with respect to ethanol intake (Fig. 5A). Post hoc comparisons between vehicle and active treatment found CaCl_2_ to significantly decrease ethanol intake the first two days of treatment (Tukey’s post hoc test: day 1 *p* = 0.006, day 2 *p* = 0.032, day 3 *p* = 0.991, day 4 *p* = 0.699, day 5 *p* = 0.891, day 6 *p* = 0.297, day 7 *p* = 0.400). The addition of NaAcamp (CaCl_2_ + NaAcamp) appeared to have a more pronounced effect compared to vehicle treatment (Tukey’s post hoc test: day 1 *p* = 0.002, day 2 *p* = 0.024, day 3 *p* = 0.220, day 4 *p* = 0.044, day 5 *p* = 0.067, day 6 *p* = 0.080, day 7 *p* = 0.020) but did not significantly enhance the ethanol-intake reducing effect of CaCl_2_. Simultaneously, water intake increased during the initial treatment period in both treatment groups (two-way ANOVA: drug treatment F_(2,40)_ = 2.43, *p* = 0.101, time F_(4.78,191)_ = 7.98, *p* < 0.001 and treatment*time interaction F_(14,280)_ = 3.77, *p* < 0.001; Tukey’s post hoc test: vehicle vs CaCl_2_ day 1 *p* = 0.004, vehicle vs CaCl_2_ + NaAcamp day 1 *p* = 0.003, vehicle vs CaCl_2_ day 2 *p* = 0.017, vehicle vs CaCl_2_ + NaAcamp day 2 *p* = 0.054, vehicle vs CaCl_2_ day 3 *p* = 0.015, vehicle vs CaCl_2_ + NaAcamp day 3* p* = 0.170; Fig. 5B). Ethanol preference decreased during the first two days of treatment compared to vehicle (two-way ANOVA: drug treatment F_(2,40)_ = 4.67, *p* = 0.015, time F_(5.73,229)_ = 7.49, *p* < 0.001 and treatment*time interaction F_(14,280)_ = 3.34, *p* < 0.001; Tukey’s post hoc test: vehicle vs CaCl_2_ day 1 *p* < 0.001, vehicle vs CaCl_2_ + NaAcamp day 1 *p* < 0.001, vehicle vs CaCl_2_ treatment day 2 *p* = 0.002, vehicle vs CaCl_2_ + NaAcamp day 2 *p* = 0.010; Fig. 5C). No significant differences in water intake or ethanol preference were detected between the different groups during the later time-points analyzed (Fig. 5B,C).

**Figure 6:**
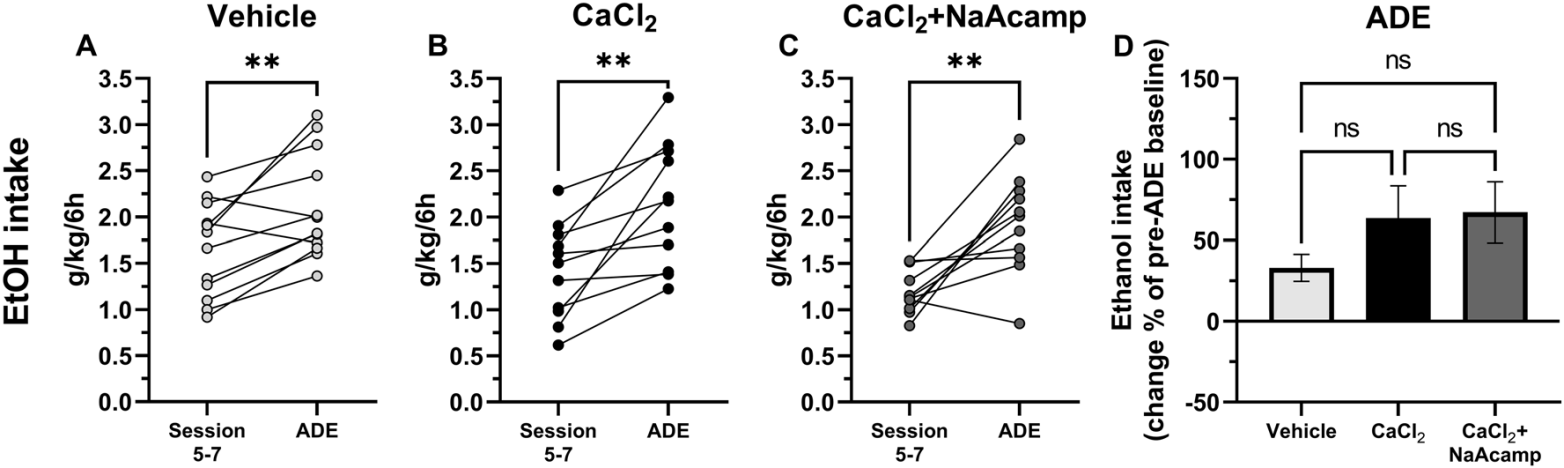
The alcohol deprivation effect (ADE) is not prevented by repeated treatment with calcium chloride or the combination of calcium chloride and sodium acamprosate. Mean ethanol intake (g/kg/6 h) of the last three sessions of the active treatment period compared with the mean of the two sessions following two weeks of ethanol deprivation (**A**–**D**). Ethanol intake significantly increased in all groups, consequently an ADE was observed in animals treated with (**A**) vehicle (NaCl 0.9%; n = 12), (**B**) CaCl_2_ (73.5 mg/kg; n = 11) and (**C**) CaCl_2_ + NaAcamp (73.5 mg/kg + 200 mg/kg; n = 11). A comparison on group level (**D**) showed no significant change in ethanol intake following alcohol deprivation between the groups. All data are presented as mean ± SEM. **p < 0.01. *ADE* alcohol deprivation effect, *CaCl*_*2*_ calcium chloride, *EtOH* ethanol, *NaAcamp* sodium acamprosate.

To assess the effect on relapse-like drinking behavior following repeated drug administration, the ADE was evaluated. Following the 2 weeks of accessing water only and on the day before reintroduction of ethanol, animals received their corresponding treatments. The second and third day of pharmacological treatment were followed by the 6-h limited access paradigm. All treatment groups (vehicle controls, CaCl_2_ injected animals or CaCl_2_ and NaAcamp injected animals) displayed an ADE (mean of two limited access sessions) and increased the ethanol intake compared with the consumption prior to alcohol deprivation (mean of last three treatment sessions) (paired *t*-test: vehicle: *t*_(11)_ = 3.69, *p* = 0.004; Fig. 6A; CaCl_2_: *t*_(10)_ = 4.00, *p* = 0.003; Fig. 6B; CaCl_2_ + NaAcamp:* t*_(10)_ = 4.05, *p* = 0.002; Fig. 6C). Further, no differences between the treatment groups were found with respect to change in ethanol intake following the alcohol deprivation period (one-way ANOVA: F_(2,31)_ = 1.387, *p* = 0.265; Fig. 6D).

## Discussion

The data presented here shows that systemic administration of sodium acamprosate (NaAcamp), which has been speculated to be inert, or calcium chloride (CaCl_2_ referred to as calcium) does not significantly increase accumbal dopamine levels per se, but sodium acamprosate in combination with calcium produces a dopamine elevating effect within the nAc. Administration of sodium acamprosate also increased nAc taurine, an effect not observed following calcium treatment. Based on this, we speculated that the combination of calcium and sodium acamprosate would produce a greater reduction in alcohol intake than what was previously demonstrated for calcium alone. Indeed, both calcium and the combination of calcium and sodium acamprosate initially reduced ethanol intake. Furthermore, the combination of calcium and sodium acamprosate visually appeared to suppress ethanol intake in a more robust manner than calcium alone, but the addition of sodium acamprosate failed to produce a significant reduction of ethanol intake compared to calcium over the treatment period. In addition, the present study found sodium acamprosate combined with calcium to prevent ADE following acute treatment, an effect that was lost after repeated exposure to the drug combination. Altogether, at a neurochemical level it appears that *N*-acetylhomotaurine exerts biological activity independently of the nature of its counter-ion, and, when administered together with calcium the effects are more pronounced. At a behavioral level calcium was found to have the predominant impact.

As AUD has a high incidence and prevalence among people in all ages, novel pharmacotherapies need to be developed. Although the pharmacological mode of action of acamprosate is and has been obscure for several decades, previous findings indicate that a dopaminergic component within the mesolimbic dopamine system is of importance^9,21,22^. In the microdialysis part of the study, the hypothesized acamprosate-induced dopamine elevation was not confirmed when analyzed over three hours after systemic administration, although a trend for an elevation was found. Further, based on recent studies showing that calcium salts possess acamprosate-like effects^12,13^ and release dopamine after local application in the nAc^15^, the hypothesis that also a systemic injection with calcium increases dopamine was revoked, although a trend for an increase to a similar extent as following sodium acamprosate was observed. Interestingly, when calcium was administered together with sodium acamprosate, the dopamine output was larger and more prolonged than observed after either agent by itself suggesting that the two substances act in concert within the mesolimbic dopamine system providing functional relevance for the previous findings following local drug administration^15^. In the present experiments systemic administration of sodium acamprosate appeared to slightly elevate dopamine, whereas no such effect was apparent following local perfusion^15^, suggesting that the modest dopamine elevating property of sodium acamprosate is not mediated through local microcircuits in the nAc. While extracellular taurine was not significantly increased by systemic calcium administration, there was a robust increase in response to sodium acamprosate as well as to the two treatments in combination. However, the increased levels of taurine were not enhanced with the addition of calcium to sodium acamprosate, suggesting that the increase of taurine is produced by *N*-acetylhomotaurine alone. Extracellular taurine and dopamine levels within the nAc have been shown to interact in response to ethanol^23,24^, an interaction that was connected to accumbal GlyRs^11,24^. Taken together, the data presented here supports previous studies, suggesting that acamprosate mimics the effects of ethanol with regard to both dopamine and taurine activation in the nAc^9,15^, and might in this neurochemical respect partially act as a substitution therapy.

The ethanol-intake reducing effect of acamprosate has successfully been demonstrated in a series of different animal models^10,14,25–28^. However, several studies in rodents have reported a progressive decline in the initial acamprosate-induced reduction in ethanol intake^17,21,22^. Furthermore, acamprosate has failed to produce a significant reduction in time to first heavy drinking day in several human trials^29–31^ raising the question of whether a decline of efficacy is also seen in some human populations. Given the present in vivo microdialysis results of the enhanced effects following co-administration of calcium and sodium acamprosate in rats, and the recently observed positive association between craving and calcium plasma concentrations in human studies^32,33^, the main question of the current voluntary ethanol consumption study was if sodium acamprosate would add on to the effect of calcium with regards to reducing ethanol intake. Although the combination of calcium and sodium acamprosate had an increased number of days with significantly decreased ethanol intake, compared to vehicle, than calcium alone, the addition of sodium acamprosate did not significantly add to the ethanol intake reducing effects of calcium over time. Thus, a greater effect of the combination appears present with respect to dopamine but was not detected in the voluntary ethanol intake paradigm. Interestingly, the combination of calcium and sodium acamprosate did not appear to lose effect over time as seen with regular acamprosate, and the intake remained decreased for a majority of the days of treatment. The clinical relevance of such a finding needs to be confirmed in future studies. It should also be noted that the animals in the microdialysis study were naïve whereas the animals in the ethanol consumption study had consumed ethanol during approximately two months where ethanol-induced alterations could influence the treatment response. However, since a previous study^22^ found acamprosate to increase dopamine following two months of ethanol consumption, tentative ethanol-induced neuroadaptations influencing the effects of acamprosate appear less likely.

Notably, at a healthy physiological condition, plasma calcium levels are tightly regulated and maintained within narrow limits^34^. However, ethanol is known to temporarily lower plasma calcium concentrations in a dose-dependent manner by decreasing secretion of parathyroid hormone and accelerating calcium excretion into the urine^35–37^. The effect of calcium treatment has been investigated and showed that increased calcium concentrations in alcohol-dependent individuals was associated with attenuation in the intensity of alcohol withdrawal symptoms^33^. Elevated plasma calcium levels were also correlated with decreased alcohol craving in dependent patients undergoing detoxification treatment^32^. In addition, plasma calcium in acamprosate treated patients has been measured, but with conflicting outcomes. On one hand, propensity to relapse did not correlate with plasma calcium levels^31,38^, but on the other hand, positive treatment outcome correlated with elevated plasma calcium levels in abstinent alcohol-dependent patients^12,39^.

As relapse to alcohol consumption is a major challenge among individuals suffering from AUD, the ADE was evaluated in the present study. While several studies have shown acamprosate to fully suppress the ethanol intake following an alcohol deprivation period in rats^12,26,40^, other studies have reported a rapid tolerance development and no reduction in ADE^17,21^. In this study, we found the combination of calcium and sodium acamprosate to inhibit the ADE following treatment in drug naïve rats whereas no attenuation of the ADE was evident for the drug combination in animals previously exposed to drug treatment. The reason for discrepancies between drug-induced influence on ADE following acute and repeated administration may be several. Perhaps the most likely explanation would be the treatment regimen, where acute or shorter periods of acamprosate treatment suppresses the ADE^12,26,40^, whereas administration for a longer period of time does not suppress the ADE as clearly^17,21^. The lack of effect on the ADE may suggest development of tolerance to the ethanol intake modifying effects of the combination of calcium and sodium acamprosate, an effect that for regular acamprosate was found to be related to a loss of ability to increase dopamine^22^. Interestingly, calcium chloride was previously demonstrated to inhibit ADE following acute administration^12^ whereas repeated administration in the present study failed to influence ADE. This would then suggest that calcium may contribute to the loss of effect over time. However, the genetic background could also have had a potential impact on the effect on ADE. We used different batches of outbred rats in the current study and genetic heterogeneity has been shown to influence both ADE as well as other types of behavior in rat lines selectively bred for alcohol-preference^41^. In human studies some attempts of genetic analysis have been forwarded in order to decrease the NNT for acamprosate^42,43^. Increased knowledge of possible genetic components in the response to acamprosate could possibly facilitate a positive treatment outcome in AUD patients. Whether tolerance develops to the ethanol-intake reducing effects of acamprosate and if this is clinically relevant or merely a rodent-specific phenomenon needs to be further explored.

In conclusion, administration of sodium *N*-acetylhomotaurine in combination with calcium chloride increases nAc taurine and produces enhanced effects on dopamine output and a robust reduction of ethanol intake in the rat. Our findings indicate that, although calcium in recent years has gained attention as the active part in acamprosate, the modest effectiveness on dopamine neurotransmission of calcium is significantly enhanced when combined with *N*-acetylhomotaurine. The present results support our previous findings^15^ that the *N*-acetylhomotaurine part of acamprosate indeed mediates pharmacological effects, but do not dismiss the value of the calcium moiety and the possibility that several mechanisms are involved to produce the drug´s beneficial effects on alcohol intake.

### Supplementary Information

Supplementary Figure 1.

Supplementary Legends.

## Data Availability

The data generated during the current study are available from the corresponding author upon reasonable request.
