# Supplementary figures and images for: Acamprosate reduces ethanol intake in the rat by a combined action of different drug components
Source: Sci Rep. 2023 Oct 19;13:17863. doi: 10.1038/s41598-023-45167-3 (PMC10587117; doi:10.1038/s41598-023-45167-3)

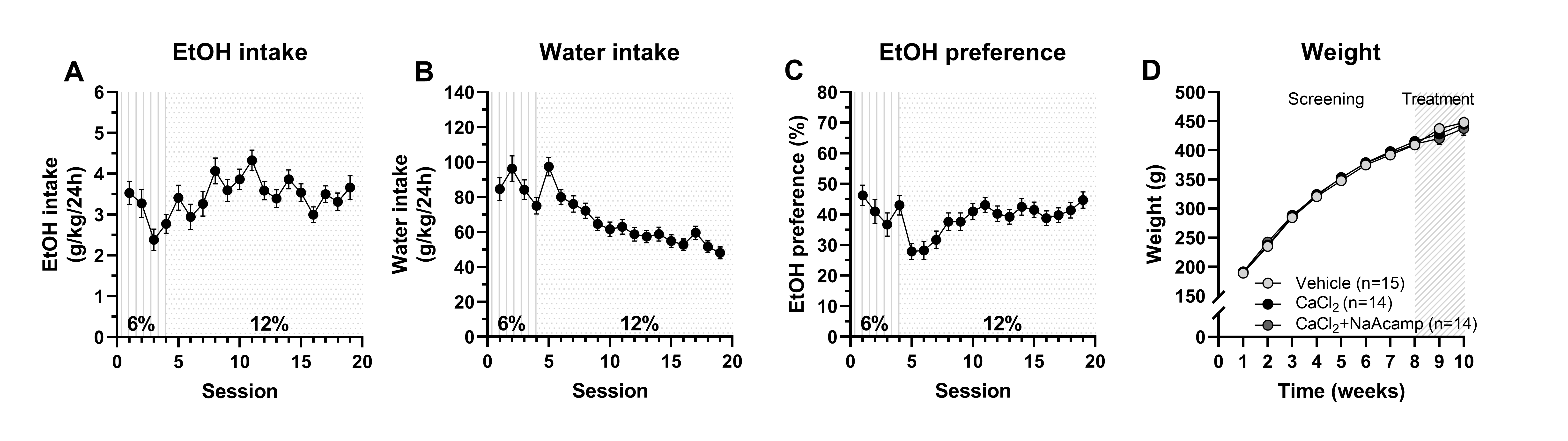

Supplement: Supplementary file 1 — Supplementary Figure 1. [file 41598_2023_45167_MOESM1_ESM.tif]
