# Supplementary material for: Acamprosate reduces ethanol intake in the rat by a combined action of different drug components
Source: Sci Rep. 2023 Oct 19;13:17863. doi: 10.1038/s41598-023-45167-3 (PMC10587117; doi:10.1038/s41598-023-45167-3)
Supplement: Supplementary file 2 — Supplementary Legends. [file 41598_2023_45167_MOESM2_ESM.docx]

**Fig. 1 Screening for voluntary ethanol intake.** Time course graphs showing **A)** ethanol intake, **B)** water intake and **C)** ethanol preference during the intermittent access (24 h) paradigm for the second voluntary ethanol consumption study. **D)** Time course graph presenting weight gain over time during screening, pharmacological treatment and alcohol deprivation revealed no significant effect by treatment. ADE=alcohol deprivation effect, CaCl_2_=calcium chloride, EtOH=ethanol, NaAcamp=sodium acamprosate.
